# A Butyrylcholinesterase Camera Biosensor Tested for Carbofuran and Paraoxon Assay

**DOI:** 10.1155/2022/2623155

**Published:** 2022-04-07

**Authors:** Miroslav Pohanka, Jitka Zakova

**Affiliations:** Faculty of Military Health Sciences, University of Defense, Trebesska 1575, Hradec Kralove CZ-50001, Czech Republic

## Abstract

Biosensors containing cholinesterase are analytical devices suitable for the assay of neurotoxic compounds. In the research on biosensors, a new platform has appeared some years ago. It is the digital photography and scoring of coloration (photogrammetry). In this paper, a colorimetric biosensor is constructed using 3D-printed multiwell pads treated with indoxylacetate as a chromogenic substrate and gold nanoparticles with the immobilized enzyme butyrylcholinesterase. A smartphone camera served for photogrammetry. The biosensor was tested for the assay of carbofuran and paraoxon ethyl as two types of covalently binding inhibitors: irreversible and pseudoirreversible. The biosensor exerted good sensitivity to the inhibitors and was able to detect carbofuran with a limit of detection for carbofuran 7.7 nmol/l and 17.6 nmol/l for paraoxon ethyl. A sample sized 25 *μ*l was suitable for the assay lasting approximately 70 minutes. Up to 121 samples can be measured contemporary using one multiwell pad. The received data fully correlated with the standard spectrophotometry. The colorimetric biosensor exerts promising specifications and appears to be competitive to the other analytical procedures working on the principle of cholinesterase inhibition. Low-cost, simple, and portable design represent an advantage of the assay of the biosensor. Despite the overall simplicity, the biosensor can fully replace the standard spectroscopic methods.

## 1. Introduction

Biosensors are analytical devices combining two crucial parts: a sensor platform known also as a physicochemical transducer and a part of biological origin that gets the specificity and selectivity to the final analysis. These devices gained popularity for low costs for manufacturing, miniaturization, saving of materials, and suitability to be used in the field of harsh conditions. Biosensors and bioassays based on the principle of digital photography have appeared in the last years as digital cameras became cheap and integrated into multifunctional devices like smartphones and tablets. This approach is based on the recording of a chemical reaction providing a color product or loss of coloration, and color depth is taken as an outputting value [1, 2]. Analytical devices and procedures like horseradish peroxidase assay using molecular imprinting technology [3], measuring of curcumin in an extract from turmeric plant [4], glycemia determination [5], and the total antioxidant capacity measurement [6] can be exampled as the relevant examples of digital camera use in a bioassay.

Cholinesterases are one of the parts of biological origin that can be used for biosensor manufacturing. Two types of cholinesterases exist, acetylcholinesterase (AChE, enzyme number EC 3.1.1.7) is the more common enzyme frequently used for biosensor construction, butyrylcholinesterase (BChE, enzyme number EC 3.1.1.8) can serve for the biosensors construction, although it is not common for this purpose. The biosensors based on cholinesterase can serve for the detection of various compounds that exert an inhibitory potency on them. Although both cholinesterases have similar specifications including the mechanism of substrate hydrolysis, some minor differences predetermine their analytical applicability. Both AChE and BChE can be inhibited by numbers of organophosphorus (e.g., nerve agents sarin soman; VX; and neurotoxic compounds like paraoxon, metrifonate, or malaoxon) and carbamate (e.g. carbofuran, rivastigmine, pyridostigmine, and neostigmine) inhibitors [7–9]. AChE can also be inhibited by various organic compounds that have only low or no affinity to BChE. Drugs and notable compounds like caffeine, tacrine, galantamine, huperzine, and donepezil can be examples [10–12]. The aforementioned carbofuran is a highly toxic insecticide with the proper chemical name 2,2-dimethyl-2,3-dihydro-1-benzofuran-7-yl methylcarbamate. Paraoxon ethyl is a neurotoxic compound with a proper chemical name O,O-Diethyl O-(4-nitrophenyl) phosphate that was formerly considered as an insecticide but withdrawn from practical use for its high toxicity. The inhibition of cholinesterase by various compounds can be easily used for analytical chemistry. Because the inhibition of the enzyme is proportional to the concentration of an inhibitor, the AChE and BChE are applicable recognition elements allowed to detect the presence of their inhibitors.

The inhibition of AChE by various compounds was used in many analytical procedures like an assay of diazinon by AChE electrochemical biosensor [13], an assay of galantamine by a microcalorimetry AChE biosensor [14], a near-infrared light fluorometric biosensor for AChE activity assay [15], and screen-printed amperometric biosensor with immobilized AChE detecting phenylmethylsulfonylfluoride [16]. The authors of the cited papers chose performance for one inhibitor, but the devices are sensitive to multiple inhibitors. Compared to AChE, the biosensors based on BChE have not gained broader interest through their applicability for practical analyses [17–20]. In this paper, a colorimetric biosensor based on BChE as a recognition element was intended as an analytical tool suitable for the detection of irreversible (organophosphorus) and pseudoirreversible (carbamate) inhibitors of cholinesterase. An original approach based on the colorimetric determination of BChE activity developed based on digital photography was chosen for biosensor construction. Simplicity combined with reliability is expected as the main benefit of the assay.

## 2. Materials and Methods

### 2.1. Standard Determination of BChE Activity in a Spectrophotometric Cuvette Assay

BChE activity was determined in a spectrophotometric cuvette assay. The same method was used for reference measuring of inhibition by testing paraoxon, ethyl, and carbofuran. The following reagents were followingly added to a disposable cuvette with an internal volume of 1.5 ml and optical length 1 cm: 550 *μ*l of phosphate-buffered saline (137 mmol/l NaCl, 2.7 mmol/l KCl, 10 mmol/l Na_2_HPO_4_ and 1.8 mmol/l KH_2_PO_4_) pH 7.4, 200 *μ*l of 5,5′-dithiobis-2-nitrobenzoic acid 0.4 mg/ml solved in saline solution (0.9% w/w NaCl in deionized water), 25 *μ*l of the tested sample (inhibitor), and 25 *μ*l of BChE solution in phosphate-buffered saline pH 7.4. The BChE had activity 10 *μ*kat/l (activity expressed with 1 mmol/l butyrylthiocholine chloride). Incubation of 10 minutes followed and 100 *μ*l of butyrylthiocholine chloride 10 mmol/l in saline solution was added. The absorbance of the arising 5-thio-2-nitrobenzoic acid was measured at 412 nm immediately and then after 120 seconds. The final activity was calculated using the extinction coefficient of 5-thio-2-nitrobenzoic acid 14,150 l × mol^−1^ × cm.

### 2.2. Preparation of Particles Modified with BChE

Gold nanoparticles with spherical shape and size under 100 nm were purchased from Sigma-Aldrich (St. Louis, Missouri, United States). The particles were chemically activated with cysteamine. A solution of cysteamine 50 mg/ml in deionized water was prepared, and 1 gram of gold nanoparticles was added to 10 ml of cysteamine solution, got to orbital shaker, and let incubate under laboratory conditions for 5 hours. The activation by cysteamine was ended by centrifugation at 9000 × *g*for 10 minutes. The supernatant was sucked out, the pellets were resuspended in 10 ml of deionized water, and the centrifugation and supernatant movements were repeated. After washing by deionized water, a 10 ml solution of 5% w/w glutardialdehyde was added to modify the particles tube with the suspension getting to the orbital spinner and left to incubate for another 5 hours and then washed by water in the same way as previously. BChE (human type, specific activity 50 units per mg protein, Sigma-Aldrich provider) solution in phosphate-buffered saline pH 7.4 with activity 10 *μ*kat/l (activity expressed to 1 mmol/l butyrylthiocholine) was added to the pelleted particles in an amount of 10 ml and let to incubate in orbital shaker for 10 hours. Finally, the mixture was centrifugated at 9000 × *g*for 10 minutes, the supernatant sucked out, and the particles were resuspended in 10 ml of phosphate-buffered saline pH 7.4. The particles were kept in the dark at 4°C until use.

### 2.3. Measuring Pad Manufacturing

**Figure 1:**
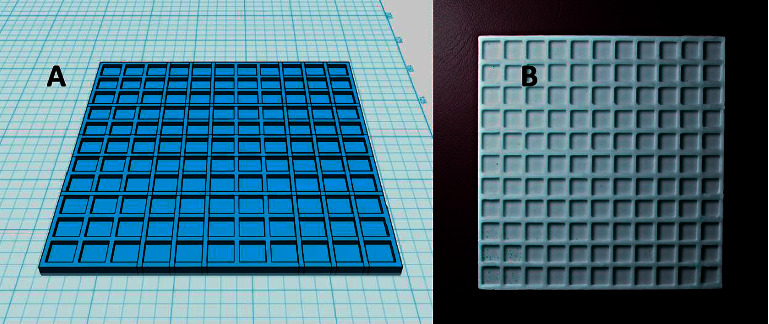
Photographs of the 3D-printed pad: project (a) and final printed pad from acrylonitrile butadiene styrene (b).

The biosensor was based on a plastic multiwell pad made by 3D printing. The pad was designed using Autodesk 123D Design (Autodesk; San Rafael, California, USA) software, and it was printed from a white acrylonitrile butadiene styrene filament with a diameter of 2.9 mm on a 3D printer Prusa i3 (Prusa Research; Prague; Czech Republic). The printer was set for nozzle temperature 285°C, bed temperature 100°C, filament loader speed 100 mm/s, and one layer thickness 0.2 mm. The final pad contained 121 wells arranged in 11 rows each of 11 wells. One well was sized 6 × 6 × 2 mm, giving a maximal volume of 72 *μ*l and an applicable volume of approximately 50 *μ*l. The pad is depicted in Figure 1.

The prepared pads were washed with 96% v/v ethanol and then with distilled water. After drying, 50 *μ*l of 50 mmol/l indoxylacetate (the concentration of indoxylacetate was a subject of optimization, see results part) solved in 96% v/v ethanol was applied per one well and left to dry in the dark under laboratory conditions. The modified pads were kept in laboratory temperature in a closed box protected from light and moisture.

### 2.4. Measuring Procedure by Biosensor

The measuring procedure started by pouring sample-sized 25 *μ*l and 25 *μ*l of the modified nanoparticles. Paraoxon ethyl and carbofuran (both analytical standards purchased from Sigma-Aldrich) were solved in deionized water served as analyzed samples. Various organic solvents were used for interference testing, and they were analyzed the same way as a standard sample. Pure deionized served in the optimization experiments as a sample. The mixture sample-modified nanoparticles were gently shaken and left to incubate for 10 minutes. After that, the whole mixture sized 50 *μ*l was injected into one well of the pretreated plates. The plate was put into a paper box 10 cm high with a hole on the upper side. The plate was photographed by a smartphone (Huawei P10; Huawei Technologies, Shenzhen, Guangdong, China) with an integrated led flash and a 10 MPx camera. The led flash was set on and activated the automatic white balance as well. An 8 bit jpeg photo was taken this way. The plate was photographed again one hour later. Each well was photographed individually to be located in the center of the photograph. Assay of a sample as well as all optimization experiments were made in five repeats according to this measuring procedure.

**Figure 2:**
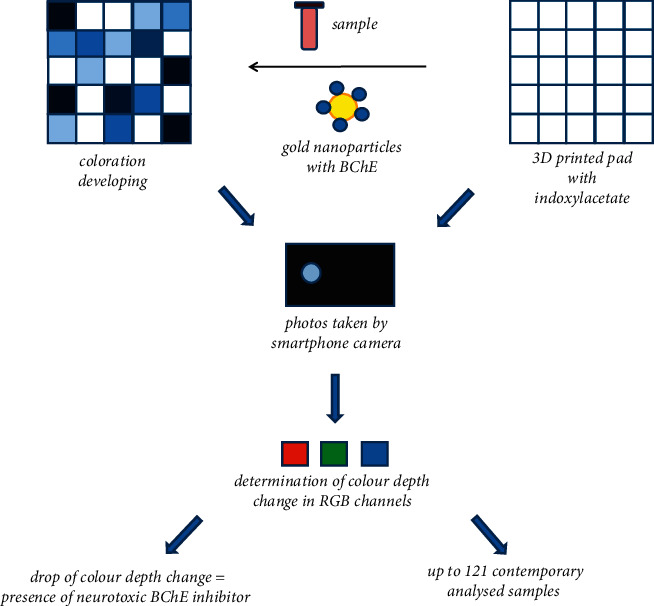
The general principle of the colorimetric biosensor assay.

Photographs collected during the measurements were processed by GIMP 2.10.28 (free and open-source software) for the determination of color depth. Five spots in the distance 2 mm from the edge of the scrutinized well were chosen, and color depths were determined for all three channels red (*R*), green (*G*), and blue (*B*). The difference of color depths before and after incubation served as an outputting signal. The general principle of the assay can be learned from Figure 2.

## 3. Results and Discussion

**Figure 3:**
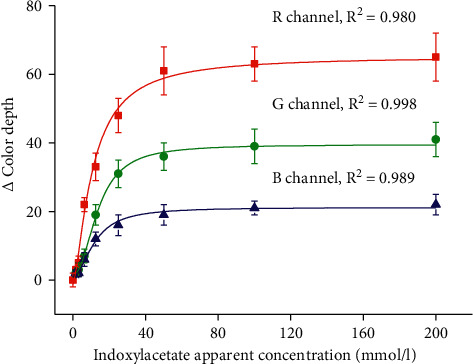
Saturation curves were received for indoxylacetate when deionized water was used as a sample for the biosensor performance. The concentration of indoxylacetate is an apparent one. The error bars indicate the standard error for five independent repeats (*n* = 5).

In the first round, the newly prepared biosensors were tested for the affinity toward the substrate. In this case, deionized water was used as a sample. Pads were prepared by application of indoxylacetate which was prepared by two-fold dilution of 200 mmol/l indoxylacetate up to the concentration 1.56 mmol/l. The three color channels were used for signal processing, and the final plot was fitted by Michaelis Menten curves. The data are depicted in Figure 3. The *R* channels appeared to be the most sensitive as the maximal theoretical color depth by Michaelis-Menten kinetics was equal to 65.3. Michaelis's constant for the *R* channel was 10.8 mmol/l, and the curve had quite a good coefficient of determination *R*^2^ equal to 0.980. The *G* channel also exerted quite good sensitivity, although worse than the *R* channel. The theoretical maximal color change for the *G* channel was equal to 39.5, and the found Michaelis constant was 13.9 mmol/l. *R*^2^ for the plot was equal to 0.998. *B* channel was less sensitive. The maximal theoretical change of color depth was equal to 21.2. Michaelis constant for the *B* channel was in the middle of values for the other channels: 12.2 mmol/l and *R*^2^ for the plot was 0.989. When considering the shape of the curves, it is obvious that the received signal was strongly dependent on the increased level of indoxylacetate. The signal was significantly increased for all of the three channels in the concentration range of indoxylacetate 1.56–25.0 mmol/l. The plateau was reached for the concentration 50 mmol/l, and further increase of indoxylacetate concentration had only minimal effect on the outputted signal. The concentration of indoxylacetate 50 mmol/l is considered optimal for the next experiments. Higher concentrations of indoxylacetate would have only minimal beneficial effect on the detected signal. The *R* channel appears to be the best for assay purposes. The fact that the *R* channel is the optimal one is not surprising. Similar finding was made in an older study [21].

Because 50 *μ*l of indoxylacetate was applied to the pad per one well, dried, and another 50 *μ*l of mixture sample-modified nanoparticles was finally applied, it can be anticipated that the concentration of indoxylacetate in the assay solution is the same as the concentration of indoxylacetate in the original solution. However, indoxylacetate is not well-soluble in water, and it is not clear whether some parts do not remain adsorbed on the pad surface. The aforementioned Michaelis constant should be considered as apparent for this reason.

**Figure 4:**
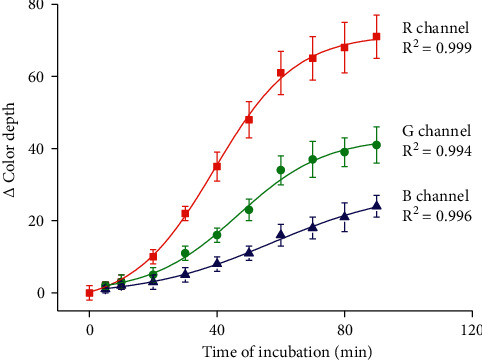
Time of incubation effect on the signal received by colorimetric biosensor performance. The error bars indicate the standard error for five independent repeats (*n* = 5).

The time necessary for signal development was optimized for indoxylacetate applied in the amount 50 mmol/l. Time intervals 0, 5, 10, 20, 30, 40, 50, 60, 70, 80, and 90 minutes were tested. Data from the experiment with the time of incubation optimization are depicted in Figure 4. The used combination of substrate concentration and enzyme activity did not provide sufficient change of color depth up to approximately 30 minutes since the reaction initialization by application of the mixture modified nanoparticles sample. The curve of the time incubation-signal was an *S-*shaped dependence. In the range 30–60 minutes an increase of signal resembling exponential growth and directing to the upper asymptote. The transition from exponential growth to upper asymptote was evident in data from the *R* and *G* channel, while the *B* channel had low sensitivity to make unambiguous conclusions. The findings are in agreement with the older experiments and the knowledge that indoxyl acetate is converted by cholinesterase with a low rate [22, 23]. Considering the experimental data, an incubation time of 60 minutes seems to be optimal for assay purposes as the upper asymptote is reached. Longer time would be beneficial for signal improving, but practical applicability of the method became reduced. Analyses lasting several hours would hardly reproducible because desiccation of solutions or degradation of reagents due to sun shining could occur. The time 60 minutes is still reasonable, and staff can manage to keep biosensors safely for such a period. Increase of sensitivity in the time above 60 minutes is marginal. The interval of 60 minutes is, therefore, a compromise, but a long time can be chosen when the significant increase of sensitivity is needed and degradation of reagents or desiccation of solutions do not represent a problem.

**Figure 5:**
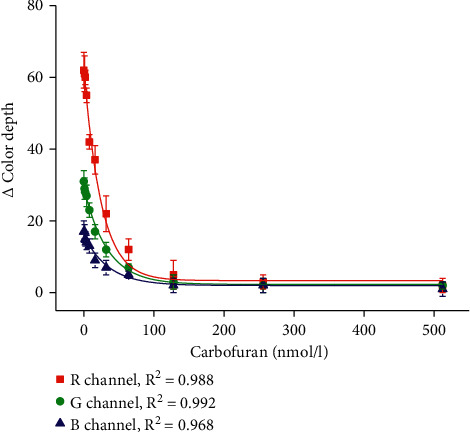
Calibration of colorimetric biosensor for carbofuran assay. The error bars indicate the standard error for five independent repeats (*n* = 5).

**Figure 6:**
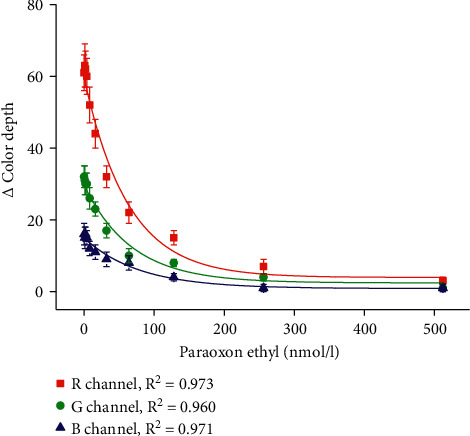
Calibration of colorimetric biosensor for paraoxon ethyl assay. The error bars indicate the standard error for five independent repeats (*n* = 5).

The biosensor was performed for the assay of carbofuran and paraoxon ethyl as two neurotoxic compounds representing pseudoirreversible (carbofuran) and irreversible (paraoxon ethyl) inhibitors of cholinesterase. Concentration range 0, 1, 2, 4, 8, 16, 32, 64, 128, 256, and 512 nmol/l was chosen for the carbofuran and paraoxon ethyl. Calibration for carbofuran is depicted in Figure 5, and calibration for paraoxon ethyl is given in Figure 6. All channels served for calibration and the limit of detection for each curve was calculated as a point on the calibration where the triplicate of standard error for the blank assay (water used as a sample) is subtracted from the average change of color depth for the blank assay. The principle of the limit of detection calculation complying with the standard rule signal to noise is equal to three for a limit of detection determination [24, 25]. Calibration of carbofuran provided a limit of detection of 7.7 nmol/l for the *R* channel, 10.6 nmol/l for calibration based on the *G* channel, and 30.5 nmol/l for the *B* channel. Limits of detection for paraoxon ethyl calibration were equal to 17.6 nmol/l for the *R* channel, 19.2 nmol/l for the *G* channel, and 52.8 nmol/l for the *B* channel. Considering the results, the tested biosensor had higher sensitivity to carbofuran than paraoxon ethyl, nevertheless both of the analytes were sufficiently detected. The *R* channel, which is the most sensitive for indigo color recording, was proved to be the best choice for an assay of neurotoxic inhibitors of cholinesterase. Regarding the limits of detection, the presented biosensor does not provide an improvement to the recently introduced biosensors for pesticides as those examples are described in the quoted papers [15, 26–28]. The colorimetric biosensor performed here does not outperform the other biosensors in the limit of detection and sensitivity but brings significant improvement in simplicity and potency to be readily manufacturable with acceptable analytical specifications.

**Figure 7:**
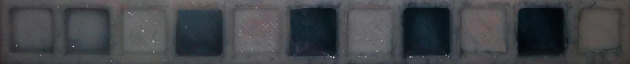
An example of assay cut of a photograph where one row composed from 11 wells is depicted. While wells 1, 3, 5, 7, 9, and 11 served for control purposes, and wells 2, 4, 6, 8, and 10 were used for an assay of carbofuran.

Although the assay was organized as an instrumental one and the change of color depth was considered as an outputting value, the coloration was quite strong and can be easily perceived by a naked eye. An example of coloration is depicted as Figure 7. The visual scoring of coloration using an etalon or even without an etalon can serve for reaction control or be an easier alternative when photogrammetry is not possible for a reason.

**Figure 8:**
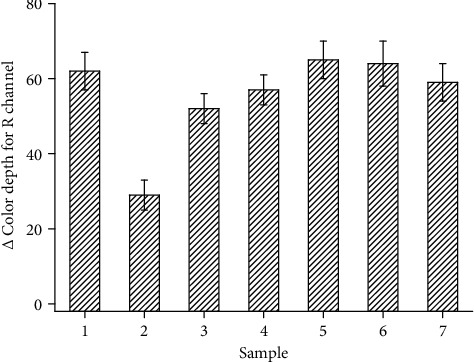
Interference testing. Sample number (1) control (deionized water), (2) ethanol 96% v/v, (3) ethanol 50% v/v, (4) ethanol 10% v/v, (5) sunflower oil, (6) phosphate-buffered saline pH 7.4, and (7) herbicide Roundup. Standard error of the mean is depicted for five independent repeats (*n* = 5).

Scrutinizing of interferences was another experiment focused on better specification of the colorimetric biosensor. Ethanol 96% v/v, ethanol 50% v/v, ethanol 10% v/v, sunflower oil, phosphate-buffered saline pH 7.4, and herbicide Roundup (Bayer, Leverkusen, Germany) served for the interference tests. The used milk, sunflower oil, and tap water come from local sources. Roundup was in the form of a solution ready to use as an herbicide. The tested chemicals were processed as any other sample, and the measured changes of color depths were compared with the change of color depth achieved when deionized water was taken as a sample. Results from the scrutinization are depicted as Figure 8. When comparing deionized water as a positive control and the other tested samples, there is obvious interference by the concentrated (96%) ethanol that caused a significant drop of detected signal with probability level 0.01 (analysis of variance, ANOVA, test). Diluted ethanol did not interfere significantly, and the other tested samples did not cause any interference as well. Considering the effect of ethanol 96% v/v, two-fold dilution of a sample prior to the assay can be a way to effectively suppress the interference of an organic solvent. The effect of an inhibitor will be reduced dramatically like the effect of solvent. Double analysis consisting of diluted and undiluted samples can provide an answer to whether drop of the signal is caused by a matrix effect or by an inhibitor. It can be expected that other organic solvents will influence the assay in the same way like ethanol when applied undiluted.

**Figure 9:**
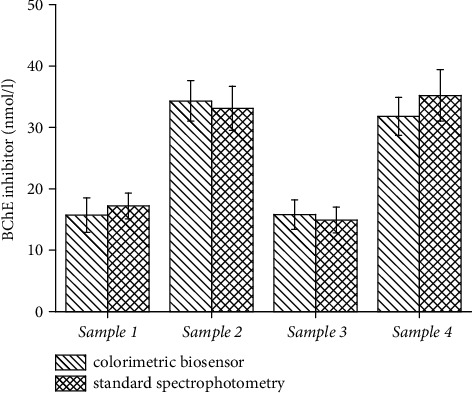
Comparison of standard spectrophotometric assays and colorimetric biosensor for measuring of cholinesterase neurotoxic inhibitor concentration. Sample 1 contained carbofuran 16 nmol/l, sample 2 carbofuran 32 nmol/l, sample 3 paraoxon ethyl 16 nmol/l, and sample 4 paraoxon ethyl 32 nmol/l. Standard error of the mean is depicted for five independent repeats (*n* = 5).

The assay by colorimetric biosensor was validated by the standard spectrophotometric cuvette assay. The same samples were analyzed by both methods. Carbofuran in the concentration 16 nmol/l and 32 nmol/l as well as paraoxon ethyl in the concentrations 16 nmol/l and 32 nmol/l served for this purpose. The concentration of the analyzed cholinesterase inhibitor was recalculated from the calibration plot for each method, and the results were compared. When compared the two methods, no significant difference was revealed at the probability levels 0.05 and 0.01 by ANOVA. Both methods can be taken for comparison regarding the ability to measure the presence of a cholinesterase irreversible or pseudoirreversible inhibitor. The assay based on the colorimetric biosensor is more suitable for routine use in the field conditions, in the conditions of point-of-care diagnosis of a poisoning source, and in other activities outside an equipped laboratory. The fact that the biosensor can contemporarily analyze up to 121 samples is another advantage. Data from the validation are depicted as Figure 9.

## 4. Conclusions

The colorimetric biosensor was proved to be a functional analytical device suitable for a simple detection of neurotoxic inhibitors of cholinesterase. It is suitable for a contemporary processing of up to 121 samples with the final result of the assay in approximately 70 minutes. The biosensor does not bring significant improvement to the other known types biosensors for inhibitors of cholinesterase assay like electrochemical [29–31] and optical [32, 33] one regarding to sensitivity or achieved limits of detection. However, here presented biosensor offers better feasibility and applicability in harsh conditions. Total easiness of the assay predetermines a wide application even outside laboratories, and analyses can be made even in harsh field conditions or any place where contamination by the neurotoxic compound can happen. Although the experiments were not focused on manufacturing issues, the biosensor were constructed in a simple manner allowing easy reproduction of the assay and introduction of it into analytical praxis.

## Data Availability

All data are inside the manuscript.
